# Assessment of body composition in Indian adults: comparison between dual-energy X-ray absorptiometry and isotope dilution technique

**DOI:** 10.1017/S0007114514001718

**Published:** 2014-08-11

**Authors:** Bharati Kulkarni, Hannah Kuper, Amy Taylor, Jonathan C. Wells, K. V. Radhakrishna, Sanjay Kinra, Yoav Ben-Shlomo, George Davey Smith, Shah Ebrahim, A. V. Kurpad, Nuala M. Byrne, Andrew P. Hills

**Affiliations:** 1 School of Exercise and Nutrition Sciences and Institute of Health and Biomedical Innovation, Queensland University of Technology, Brisbane, QLD, Australia; 2 Clinical Division, National Institute of Nutrition, Jamai Osmania PO, Hyderabad500 007, India; 3 Department of Non-communicable Disease Epidemiology, London School of Hygiene and Tropical Medicine, London, UK; 4 School of Social and Community Medicine, University of Bristol, Bristol, UK; 5 Childhood Nutrition Research Centre, UCL Institute of Child Health, London, UK; 6 South Asia Network for Chronic Disease, Public Health Foundation of India, New Delhi, India; 7 Saint John's Research Institute, Bangalore, India; 8 Mater Mothers' Hospital, Mater Research Institute – University of Queensland and Griffith Health Institute, Griffith University, Brisbane, QLD, Australia

**Keywords:** Body composition, Dual-energy X-ray absorptiometry, Isotope dilution technique, Indian adults, Bland–Altman analysis

## Abstract

Dual-energy X-ray absorptiometry (DXA) and isotope dilution technique have been used as reference methods to validate the estimates of body composition by simple field techniques; however, very few studies have compared these two methods. We compared the estimates of body composition by DXA and isotope dilution (^18^O) technique in apparently healthy Indian men and women (aged 19–70 years, *n* 152, 48 % men) with a wide range of BMI (14–40 kg/m^2^). Isotopic enrichment was assessed by isotope ratio mass spectroscopy. The agreement between the estimates of body composition measured by the two techniques was assessed by the Bland–Altman method. The mean age and BMI were 37 (sd 15) years and 23·3 (sd 5·1) kg/m^2^, respectively, for men and 37 (sd 14) years and 24·1 (sd 5·8) kg/m^2^, respectively, for women. The estimates of fat-free mass were higher by about 7 (95 % CI 6, 9) %, those of fat mass were lower by about 21 (95 % CI − 18, − 23) %, and those of body fat percentage (BF%) were lower by about 7·4 (95 % CI − 8·2, − 6·6) % as obtained by DXA compared with the isotope dilution technique. The Bland–Altman analysis showed wide limits of agreement that indicated poor agreement between the methods. The bias in the estimates of BF% was higher at the lower values of BF%. Thus, the two commonly used reference methods showed substantial differences in the estimates of body composition with wide limits of agreement. As the estimates of body composition are method-dependent, the two methods cannot be used interchangeably.

Estimation of body composition is a vital element of nutritional assessment as fat and fat-free compartments of body mass have different health implications. Fat mass (FM) is closely linked with metabolic complications of obesity because the adipose tissue functions as an endocrine organ that releases bioactive substances having pro-inflammatory properties^(^ ^1^ ^)^. In contrast, fat-free mass (FFM), especially muscle mass, plays a protective role against the risk of chronic diseases including diabetes and osteoporosis^(^ ^2^ ^)^. Ethnic differences in the relationship between BMI and disease risk have been associated with differences in body composition^(^ ^3^ ^,^ ^4^ ^)^.

A number of techniques are available for the assessment of body composition, and the choice of technique usually depends on precision, accuracy, ease of application as well as the cost. DXA is increasingly used for body composition assessment because of its high precision and low dose of radiation. A number of studies have validated other, less precise, techniques such as anthropometry and bioelectrical impedance analysis against DXA as a reference method^(^ ^5^ ^–^ ^7^ ^)^. However, DXA is not without limitations. Although studies have shown that estimates of body composition by DXA are highly correlated with those derived using more accurate methods, variations have been reported between the estimates^(^ ^8^ ^,^ ^9^ ^)^.

With increasing recognition of the association between the high prevalence of the metabolic syndrome and ‘thin-fat’ phenotype in Indians, there is enhanced interest in the assessment of body composition^(^ ^10^ ^,^ ^11^ ^)^. A number of studies in India have reported the body composition of different population groups using different techniques including DXA^(^ ^12^ ^–^ ^15^ ^)^. However, different studies that have compared the estimates of body composition using different methods of body composition measurement need to consider the variation in estimates associated with these methods. Moreover, studies comparing different methods of body composition measurement tend to be population-specific due to ethnic variations in body composition^(^ ^16^ ^)^. Studies comparing the estimates of body composition using DXA with those measured by other reference methods have not so far been reported in India. Therefore, the aim of the present study was to compare the estimates of body composition by DXA with those using the isotope dilution technique.

## Participants and methods

The present study was conducted according to the guidelines laid down in the Declaration of Helsinki, and all procedures involving human participants were approved by the ethics committees of the National Institute of Nutrition, Hyderabad, India, the London School of Hygiene & Tropical Medicine, UK and Queensland University of Technology, Australia. Written informed consent was obtained from all participants.

### Participants

Healthy volunteers aged 19–70 years were enrolled in the present study from two pre-established cohorts (Andhra Pradesh Children and Parents Study (APCAPS), *n* 58 and Indian Migration Study (IMS), *n* 94) living around the city of Hyderabad, India. The APCAPS cohort was established to assess the long-term impact of early nutrition supplementation provided to pregnant women and young children^(^ ^17^ ^)^, whereas the IMS cohort was established to examine the association between rural to urban migration and cardiometabolic risk^(^ ^18^ ^)^. To obtain a representative sample, participants were chosen on the basis of pre-defined age, sex, cohort, intervention group (in the case of the APCAPS) or rural/urban migrants (in the case of the IMS), and BMI categories (see online supplementary Tables S1 and S2). The target enrolment was 160 participants.

### Demographic and anthropometric data

Demographic information was collected from all study participants using an interviewer-administered questionnaire. Weight was measured to the nearest 0·1 kg in light clothing without footwear, using a digital Seca scale (www.seca.com). Standing height was measured to the nearest 1 mm using a portable stadiometer (Leicester Height Measure; Chasmors Limited). Anthropometric measurements were taken twice, and the average of the two values for each measurement was used in the analysis. BMI was calculated as weight (kg)/height (m^2^).

Body composition of each participant was assessed by DXA and isotope dilution technique on the same day.

### Isotope dilution technique

Participants arrived at the National Institute of Nutrition in the morning after an overnight fast. A baseline urine sample was collected on arrival for the measurement of background isotopic enrichment, followed by the administration of an oral dose of ^18^O (0·2 g/kg body weight) to each participant at about 09.00 hours. The bottle containing the dose was rinsed with 50 ml deionised water before its consumption by the participants. A light breakfast was provided with 50 ml water at about 10.00 hours. Any subsequent oral intake was avoided. Follow-up urine samples were collected 4 and 5 h after the intake of dose to allow complete equilibration of the isotope within the body water compartments^(^ ^19^ ^)^. Aliquots of all the urine samples were stored in screw-capped glass containers at − 20°C until analysis. Isotopic enrichment in the pre- and post-dose urine samples, the dose given and the local tap water was measured using isotope ratio mass spectrometry (Hydra 20-20; SerCon) at St John's Research Institute, Bangalore, India. The CV calculated using repeated analysis for the natural background samples as well as for the enriched samples was less than 0·01 %. Each sample was analysed in duplicate, and the mean was used for the analysis. Total body water was calculated, allowing a correction by 0·7 % for *in vivo* exchange^(^ ^20^ ^)^. FFM was calculated from total body water using a hydration constant of 0·73. FM was calculated by subtracting FFM from body weight.

### Dual-energy X-ray absorptiometry scans

Body composition was assessed by a whole-body DXA scan using a fan-beam DXA machine (Hologic Discovery A model, software version 12.5; www.hologic.com). The scanner was calibrated periodically with a phantom, and its performance was monitored according to the manufacturer's quality assurance protocol. During the scan, the participants were asked to lie supine on the scanning bed with their arms at their sides. Standard software options were used to calculate the total FFM and FM. FFM was the sum of lean soft tissue mass and bone mineral content. Precision estimates (CV%) of body composition by DXA based on repeat measurements in thirty participants were 0·7 and 1·4 % for FFM and FM, respectively.

### Statistical analyses

All analyses were conducted using Stata (version 11.2; StataCorp). As FFM and FM showed a skewed distribution, these variables were log-transformed before analysis, and, therefore, the mean differences between the two are expressed as ratios. Other continuous variables were used in the original scale. Differences between the body composition estimates (FFM, FM and BF%) by DXA and isotope dilution technique were assessed using paired *t* tests. The Bland–Altman method was used to assess the agreement between the estimates of body composition determined by the two techniques^(^ ^21^ ^)^. The mean difference in the estimates by the two techniques (bias) and their 95 % limits of agreement (2 sd of the mean difference) were calculated. As the bias and limits of agreement for FFM and FM were on a logarithmic scale, these values are presented as ratios. Correlation coefficients were calculated to examine the association between the average values of body composition measurements by the two methods and the difference between these methods, which indicates the proportional bias. All analyses were conducted for the whole sample and additionally stratified by sex.

## Results

**Fig. 1:**
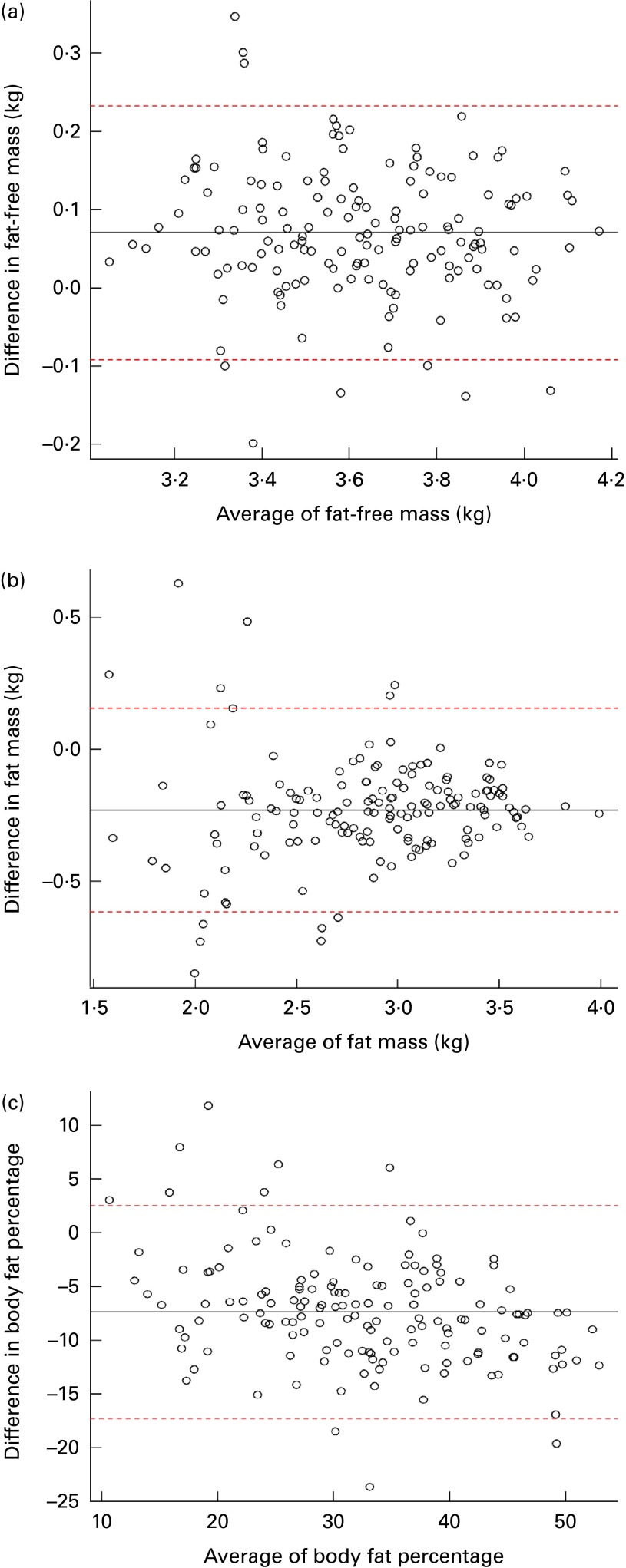
Bland–Altman plot of the estimates of (a) fat-free mass, (b) fat mass and (c) body fat percentage by dual-energy X-ray absorptiometry and isotope dilution technique. Values of fat-free mass and fat mass are presented on a logarithmic scale. The central dashed line represents the mean difference between the measures. The upper and lower dashed lines represent the 95 % limits of agreement (2 sd of the mean difference). (A colour version of this figure can be found online at http://www.journals.cambridge.org/bjn).

A total of seventy-three men and seventy-nine women participated in the study. Their characteristics are presented in Table 1. The participants were chosen to represent a wide range of BMI varying from 13·8 to 39·7 kg/m^2^. The total mass value measured by DXA showed a strong correlation with weight measured by the scale (0·99, *P*< 0·01). Although there was a strong correlation between the estimates of body composition measured by DXA and isotope dilution technique (FFM: *r* 0·95, FM: *r* 0·95, BF%: *r* 0·89 all *P*< 0·01), the estimates of FFM obtained by DXA were higher than those obtained by the isotope dilution technique in the whole sample as well as in the subgroups stratified by sex (Table 2). The estimates of FM and BF% obtained by DXA were lower than those measured by the isotope dilution technique. On average, DXA overestimated the FFM values by about 7 (95 % CI 6, 9 %) % compared with the isotope dilution technique (Table 3; Fig. 1(a)). However, the limits of agreement showed that 95 % of the estimates of FFM measured by DXA were expected to be between 9 % lower and 26 % higher than the values measured by the isotope dilution technique. For FM, the bias was greater, and, on average, the estimates by DXA were about 21 % lower than those by the isotope dilution technique (Table 3; Fig. 1(b)). The limits of agreement for FM were much larger ( − 54 to 17 %) than those for FFM between the two methods. There was no correlation between the bias and the average values of the estimates measured by the two methods for both FFM and FM, indicating that the bias in the estimates of FFM and FM did not change with the amount of FFM and FM, respectively. On average, the estimates of BF% measured by DXA were lower than those measured by the isotope dilution technique by 7·4 (95 % CI − 8·2, − 6·6 %) % (Table 3; Fig. 1(c)). The bias in the estimates of BF% was negatively correlated with the average values of BF%, indicating that the difference between the two methods was higher for the participants with lower values of BF% (Table 3). The estimates of FFM, FM and BF% measured by DXA explained about 89, 85 and 78 % of the variation in the respective estimates measured by the isotope dilution technique.

**Table 1:** Characteristics of the study participants (Mean values and standard deviations)

	Men (*n* 73)	Women (*n* 79)
	Mean	sd	Minimum	Maximum	Mean	sd	Minimum	Maximum
Age (years)	37	15	19	70	37	14	19	62
BMI (kg/m^2^)	23·3	5·1	14·5	37·6	24·1	5·8	13·8	39·7
Height (cm)	165·5	6·3	149·1	183·2	151·7	5·6	136·0	162·5
Weight (kg)	64·1	15·1	38·7	108·0	55·6	14·3	31·2	103·7
TM by DXA (kg)	64·0	15·0	39·2	107·6	55·7	14·2	31·4	102·6

TM, total mass; DXA, dual-energy X-ray absorptiometry.

**Table 2:** Estimates of body composition by dual-energy X-ray absorptiometry (DXA) and isotope dilution technique (Mean values and standard deviations)

	*n*	Isotope dilution technique	DXA	*P* *
Mean	sd	Mean	sd
Fat-free mass (kg)						
Whole sample	152	37·42	9·45	40·09	9·84	< 0·01
Men	73	44·18	7·98	46·89	8·28	< 0·01
Women	79	31·17	5·65	33·79	6·39	< 0·01
Fat mass (kg)						
Whole sample	152	22·27	10·20	17·78	8·3	< 0·01
Men	73	19·93	9·58	15·09	7·49	< 0·01
Women	79	24·43	10·34	20·27	8·28	< 0·01
Body fat percentage						
Whole sample	152	36·3	10·9	28·9	9·2	< 0·01
Men	73	29·8	8·7	22·3	6·6	< 0·01
Women	79	42·3	9·1	35·1	6·6	< 0·01

* *P* value was obtained from the paired *t* test of the difference.

**Table 3:** Bias and 95 % limits of agreement for measures of body composition by dual-energy X-ray absorptiometry (DXA) compared with the isotope dilution technique

	*n*	Bias*	95 % CI	Limits of agreement†	*r* ‡	*P* §
Fat-free mass (kg)						
Whole sample	152	1·07	1·06, 1·09	0·91, 1·26	− 0·077	0·35
Men	73	1·06	1·04, 1·08	0·92, 1·23	− 0·127	0·28
Women	79	1·08	1·06, 1·10	0·91, 1·29	0·083	0·47
Fat mass (kg)						
Whole sample	152	0·79	0·77, 0·82	0·54, 1·17	0·045	0·58
Men	73	0·75	0·71, 0·79	0·48, 1·17	0·043	0·71
Women	79	0·84	0·81, 0·86	0·63, 1·12	− 0·181	0·11
Body fat percentage						
Whole sample	152	− 7·4	− 8·2, − 6·6	− 17·3, 2·6	− 0·345	< 0·01
Men	73	− 7·5	− 8·7, − 6·3	− 17·7, 2·8	− 0·428	< 0·01
Women	79	− 7·3	− 8·4, − 6·2	− 17·0, 2·4	− 0·513	0·03

* Mean bias and 95 % CI for fat-free mass and fat mass are expressed as the ratio of DXA:isotope dilution technique values. Bias is the difference (DXA − isotope dilution) between the log-transformed values of fat-free mass and fat mass estimated from the two techniques. The values of body fat percentage are given on the original scale.

† 95 % Limits of agreement (2 sd of the mean difference) expressed as the ratio of DXA:isotope dilution values of fat-free mass and fat mass. The values of body fat percentage are given on the original scale.

‡ *r* is Pearson's correlation coefficient between the difference between DXA and isotope dilution technique and the average of DXA and isotope measures of fat-free mass, fat mass and body fat.

§ Significance of the correlation coefficient.

## Discussion

The present study compared the estimates of body composition measured by two precise techniques – DXA and isotope dilution technique – in apparently healthy, weight-stable Indian men and women with a wide range of BMI. In this sample of participants, the estimates of FFM were higher whereas those of FM and BF% were lower using DXA than using the isotope dilution technique. The agreement between the two methods was not good as indicated by the significant bias between these methods and wide limits of agreement, especially for the estimates of FM and BF%. The bias in the estimates of BF% measured by the two methods was higher for individuals with lower values of BF%. The present study indicates that these two methods cannot be used interchangeably as systematic differences exist between the estimates of body composition.

Previous studies that have compared the estimates of body composition by DXA and isotope dilution technique have reported inconsistent results. In general, studies that used older equipment (e.g. Hologic QDR 2000, Hologic QDR 1000W, Lunar DPX-L) with scans done in a pencil-beam mode have shown underestimation of FFM and overestimation of FM and BF% by DXA compared with the isotope dilution technique^(^ ^22^ ^,^ ^23^ ^)^. In contrast, studies that used newer equipment (e.g. Hologic QDR 4500W, QDR 4500A) have shown overestimation of FFM by DXA compared with the isotope dilution technique^(^ ^9^ ^,^ ^24^ ^)^. For example, a study in Chinese women in 1999 has shown that DXA (Hologic QDR 2000) underestimated FFM by 0·5 kg and overestimated BF% by 0·8 %^(^ ^23^ ^)^. Similarly, a study from the UK (*n* 28) in 1992 has also found that DXA (Hologic QDR 1000W) underestimated FFM by 0·2 kg compared with the isotope dilution technique^(^ ^22^ ^)^. However, a later study by Deurenberg-Yap & Deurenberg^(^ ^24^ ^)^ in Chinese, Malays and Indians living in Singapore has shown overestimation of FFM and underestimation of BF% by DXA (Hologic QDR 4500W) compared with the ^2^H dilution technique. Similarly, a study by Schoeller *et al.* ^(^ ^9^ ^)^ from the USA that compared body composition by DXA with other reference techniques in 1195 men and women (DXA compared with the isotope dilution technique in 395 participants) has also shown that DXA overestimated FFM by 1·8 to 4·7 kg and underestimated FM by about 1·3 to 5·1 kg. The findings of the present study that used a newer model of DXA (Hologic Discovery) are consistent with relatively recent studies that have shown overestimation of FFM by DXA compared with the isotope dilution technique. However, the magnitude of bias in the estimates of FFM (approximately 3 kg) and FM (approximately 4·5 kg) in the present study is larger than the bias reported in other studies.

A number of studies (Table 4) comparing the estimates of body composition by DXA with those by multi-component criterion methods have also reported inconsistent results^(^ ^22^ ^,^ ^25^ ^–^ ^29^ ^)^. Although the majority of these studies reported underestimation of BF% by DXA, similar to the present study, a few studies have reported a bias in the opposite direction. For example, a study by Williams *et al.* ^(^ ^30^ ^)^ compared DXA with a four-compartment (4C) model and reported the overestimation of FM and BF% by DXA in non-obese adults. In contrast, a few studies did not detect significant difference in BF% by DXA compared with the 4C model^(^ ^31^ ^–^ ^33^ ^)^.

**Table 4:** Comparison of body fat percentage (BF%) measured by dual-energy X-ray absorptiometry (DXA) and the four-compartment model (4C) (Mean values and standard deviations)

Study†		Age		Type of the X-ray beam	Mean difference in BF% (4C − DXA)
Sex	Mean	sd	DXA system
Bergsma-Kadijk *et al.* ^(^ ^26^ ^)^	20 F	22	2	GE Lunar DPX	Pencil	3·1*
	18 F	72	4			5·3*
Prior *et al.* ^(^ ^32^ ^)^	91 M	21	2	Hologic QDR	Pencil	− 0·6
	81 F			Hologic QDR 1000W		− 0·2
Withers *et al.* ^(^ ^29^ ^)^	24 M	Athletes: 22	5	GE Lunar DPX-L	Pencil	Athletes: 3·5*
		Non-athletes: 25	5			Non-athletes: 1·3*
	24 F	Athletes: 24	6			Athletes: 1·3*
		Non-athletes: 22	3			Non-athletes: 0·4
Arngrimsson *et al.* ^(^ ^25^ ^)^	22 M	Athletes: 21	2	Hologic QDR 1000W	Pencil	Athletes: 2·9*
		Non-athletes: 21	3			Non-athletes: 2·9*
	22 F	Athletes: 21	2			Athletes: 4·0*
		Non-athletes: 21	3			Non-athletes: 2·2*
Deurenberg-Yap *et al.* ^(^ ^27^ ^)^	144 M	42	13	Hologic QDR 4500	Fan	3·8 %*
	147 F	36	12			2·3 %*
Van Der Ploeg *et al.* ^(^ ^28^ ^)^	118 M	31	12	GE Lunar DPX-L	Pencil	1·9 %*
	34 F	26	8			1·7 %*
van Marken Lichtenbelt *et al.* ^(^ ^31^ ^)^	27 M	32	6	GE Lunar DPX-L	Pencil	− 0·8
Williams *et al.* ^(^ ^30^ ^)^	26 M	20	1	GE Lunar	Narrow	− 1·7 %*
	44 F	20	1	Prodigy	Fan	− 2·0 %*
LaForgia *et al.* ^(^ ^33^ ^)^	8 M	36	13	GE Lunar Prodigy	Narrow	0·3 %
	6 F				Fan	

F, female; M, male.

* Statistically significant difference between DXA and the 4C model (*P*< 0·05).

† Includes weight-stable, healthy adults.

Differences in the results of studies comparing the estimates of body composition by DXA with those by other techniques could be related to a number of factors such as the use of DXA machines by different manufacturers and differences in the scan mode or software used for analyses. Machines developed by different manufacturers as well as different models by the same manufacturer, although based on the same physical principles, differ in various aspects such as the generation of high- and low-energy X-ray beams, X-ray detectors, calibration methodology, algorithms used for selective tissue imaging, edge detection, region-of-interest definition, system calibration, etc.^(^ ^34^ ^)^. Variations in the estimates of body composition with the machines developed by different manufacturers and even with different models by the same manufacturer have been reported^(^ ^35^ ^–^ ^38^ ^)^. In addition, isotope dilution technique has a number of limitations as the estimates of body composition are based on a number of assumptions including the equal distribution of a tracer in body water and constant hydration of FFM^(^ ^39^ ^)^. Both these techniques are thus error prone, and a lack of agreement between the methods for the estimation of body composition could be related to a number of factors that can lead to inaccuracies in the estimates.

However, limits of agreement between the two methods observed in the present study were wider (FFM: − 9,+26 %; FM: − 46,+17 %; BF%: − 17·3, 2·6 %) than those reported by other studies, the majority of which have reported the limits of agreement between ± 10 % of the mean^(^ ^40^ ^)^. In contrast, a few other studies have reported that DXA could under- or overestimate the FM of an individual by almost 28 %^(^ ^22^ ^)^. One of the reasons for the narrow limits of agreement reported by other studies could be the exclusion of extreme values of the differences. For example, Schoeller *et al.* ^(^ ^9^ ^)^ excluded observations in which the difference in the estimates of FFM measured by DXA and isotope dilution technique was >6 kg. The present study did not exclude observations with larger differences between the measurements, which may have contributed to a larger bias between the measurements reported herein.

An interesting finding of the present study is that the bias in the estimates of BF% by the two methods was higher at lower values of BF% (*r* − 0·345, *P <*0·001; Table 3). A previous study comparing the estimates of abdominal fat by DXA with those using MRI in this sample has also shown that overestimation of abdominal fat by DXA was greater in individuals with less abdominal fat^(^ ^41^ ^)^. It is possible that the algorithms used for the estimation of body composition by DXA produce a larger error at very low levels of body fat. A number of studies from other centres have shown that the bias in the estimates of body composition by DXA varied according to a number of factors including age, body size, body fat, sex, health status, type of the instrument, etc.^(^ ^30^ ^)^.

An important strength of the present study includes enrolment of a large sample representing a broad range of age and BMI. In addition, the present study used ^18^O as the isotope tracer that may provide a more accurate estimate of total body water than a more commonly used ^2^H_2_O as ^18^O exchanges to a smaller degree with non-aqueous molecules^(^ ^39^ ^)^. As differences in body composition in relation to ethnicity are well known, population-specific validation studies comparing DXA with other precise methods are required. Therefore, the present study provides much-needed evidence on the comparability of DXA with the isotope dilution technique in a population group that is known to have a high percentage of body fat at a given BMI compared with other ethnic groups^(^ ^10^ ^,^ ^15^ ^)^. A limitation of the present study is the use of the isotope dilution technique for validating DXA measurements of body composition instead of a multi-component criterion technique. However, estimates of body composition using the isotope dilution technique are highly correlated with those using the criterion technique of the 4C model^(^ ^27^ ^)^. A study comparing the estimates of body composition by densitometry, DXA and isotope dilution technique with those by the 4C model in Asian adults has shown that estimates of BF% by the isotope dilution technique had the lowest bias while those by DXA had the highest bias compared with the 4C model, suggesting that the isotope dilution technique may be the best two-compartment model for measuring body fat^(^ ^24^ ^)^.

In conclusion, the present study shows that estimates of body composition by two commonly used reference methods such as DXA and isotope dilution technique may be considerably different at the individual level, with particularly larger differences in the estimates of BF%. The two methods are therefore not directly interchangeable. However, the differences in the absolute values at the individual level may not affect the results of the studies exploring the relationship of body composition using either of these methods with health outcomes, as these values were highly correlated. Additional studies are required to develop correction factors that could be used to calibrate DXA in order to alleviate the differences in these two methods.

## Supplementary material

To view supplementary material for this article, please visit http://dx.doi.org/10.1017/S0007114514001718
